# Far from the Eyes, Close to the Heart: Dysbiosis of Gut Microbiota and Cardiovascular Consequences

**DOI:** 10.1007/s11886-014-0540-1

**Published:** 2014-10-11

**Authors:** Matteo Serino, Vincent Blasco-Baque, Simon Nicolas, Remy Burcelin

**Affiliations:** 1 Institut National de la Santé et de la Recherche Médicale (INSERM), Toulouse, France; 2 Université Paul Sabatier (UPS), Unité Mixte de Recherche (UMR) 1048, Institut de Maladies Métaboliques et Cardiovasculaires (I2MC), 31432 Toulouse CEDEX 4, France; 3 Faculté de Chirurgie Dentaire de Toulouse, Université Paul Sabatier, 3, chemin des Maraîchers, 31062 Toulouse CEDEX, France

**Keywords:** Gut microbiota, Cardiovascular diseases, High-fat diet, Metagenomics, Inflammation, Protein-rich diet

## Abstract

These days, the gut microbiota is universally recognized as an active organ that can modulate the overall host metabolism by promoting multiple functions, from digestion to the systemic maintenance of overall host physiology. Dysbiosis, the alteration of the complex ecologic system of gut microbes, is associated with and causally responsible for multiple types of pathologies. Among the latters, metabolic diseases such as type 2 diabetes and obesity are each distinguishable by a unique gut microbiota profile. Interestingly, the specific microbiota typically found in the blood of diabetic patients also has been observed at the level of atherosclerotic plaque. Here, we report evidence from the literature, as well as a few controversial reports, regarding the putative role of gut microbiota dysbiosis-induced cardiovascular diseases, such as atherosclerosis, which are common comorbidities of metabolic dysfunction.

## Introduction

Animals and microorganisms have coevolved to form a complex superorganism to benefit each other with functions they have not evolved on their own. For example, whereas microorganisms digest complex carbohydrates from plants and synthesize vitamins, the human host provides nutrients from alimentation and a protective environment. Excitingly, microbial cells largely outnumber eukaryotic cells. For instance, in humans, microbial cells are estimated to be 10 times more numerous than eukaryotic cells [1]. Moreover, the overall microbial genome—the *metagenome—*has been shown to be organized in about 100 times more genes than our human genome [2••], despite a molecular complexity, in terms of base pairs, one order of magnitude lower than that of human DNA. Furthermore, microbial gene richness is under the control of dietary intervention, and low microbial gene diversity is prognostic of increased susceptibility to metabolic alterations [3••].

For years, the role of the gut microbiota was thought to be relegated merely to digestive function. Nowadays, however, the gut microbiota is widely recognized as an active organ that can modulate multiple functions of the host, from development of the intestinal immune system to hepatic and energy metabolism and to modulation of the brain in terms of behavior development and motor activity [4].

By means of germ-free (also called *axenic*) murine models, some light has been shed on the mechanisms by which the gut microbiota modulates host metabolism with regard to diet-induced obesity [5], energy metabolism [6], and maintenance of intestinal physiology [7]. These mice in fact are completely sterile from a microbial point of view, and their lack of gut microbiota determines a multitude of both structural and functional alterations [8]. Hence, colonization (transplantation) of axenic mice with gut microbiota issued from other animal models (e.g., obese mice [6]) or from human stools (termed *humanization* [9, 10]) may allow identification of metabolic processes and the related molecular mechanisms under the control of intestinal microbes. Furthermore, we and others have shown that targeting intestinal microbiota via dietary treatment [11], dietary fiber [12•], or antibiotics [13, 14] ameliorated metabolic features such as glucose tolerance, insulin sensitivity, and body weight gain and reduced the associated chronic low-grade inflammation [15].

Our knowledge of the ecologic organization of the gut microbiota and its alteration—dysbiosis—has increased thanks to advances in high-throughput molecular sequencing [16], which obviates the need for laboratory cultivation to identify bacteria. Today, the so-called holistic “-omics” approach allows very detailed analysis of the gut microbiota at the level of both taxonomy, identifying the *microbiota* (phyla to species), and function, identifying the *microbiome* (gene repertoire) [16]. For example, the Firmicutes and Bacteroidetes are recognized as the two major bacterial phyla populating gut microbiota, up to 90 %.

Dysbiosis of the gut microbiota has been reported for several pathologies, such as inflammatory chronic diseases of the intestine [17], intestinal cancer [18], and susceptibility to allergy [19], and especially for metabolic diseases, such as type 2 diabetes and obesity [20]. For example, the obesity component appears to set the tone for gut microbial dysbiosis, because any imbalance favoring the Firmicutes has been identified as the “bacterial signature” of obesity in humans [21] as well as in mice [22]. On the other hand, lack of obesity in type 2 diabetic patients is characterized by Bacteroidetes dominance [23].

Metabolic diseases are well-known common risk factors for development of severe cardiovascular diseases (CVDs) [24], such as atherosclerosis, stroke, and myocardial infarction. In the past decade, there has been a global increase in CVD-induced death, mainly as the result of coronary heart disease (7.6 million) and stroke (5.7 million) [25]. The World Health Organization estimates about 20 million CVD deaths in 2015, rendering CVD as the major contributor to mortality worldwide. If we take into account only the data relative to the USA, the situation resembles a war bulletin. In fact, it is estimated that 3.88 % of the US population older than 18 years will have a stroke by 2030. From 2010 to 2030, the total direct annual medical cost attributable to stroke is expected to increase from $71.55 billion to $183.13 billion, with an overall estimated increase of 129 % [26].

Recent evidence from the literature provides a link between microbial activity and the modulation of overall cardiometabolism. These experimental results were obtained through metabolomics [27], the overall analysis of plasma metabolites (metabolome), which enabled identification of key atherogenic microbial products, such as trimethylamine (TMA) and its hepatic-generated metabolite TMA-*N*-oxide (TMAO), resulting from microbial metabolism of dietary nutrients such as choline and L-carnitine [28••].

However, the impact of gut microbiota and its dysbiosis on the induction of CVD remains a subject for debate because of some controversial aspects of the molecular mechanisms underlying this relationship, which are discussed in this review.

## The Role of Gut Microbiota in Cardiovascular Impairment

At the end of the 1990s, the link between gut microbiota and CVD had not yet been discovered, and the first article to focus on this relationship failed to demonstrate any interaction. In this context, Wright et al. [29] reported that axenic (germ-free) ApoE knock-out mice were not protected from the development of atherosclerotic plaque, suggesting that the gut microbiota is not mandatory for the etiology of atherosclerosis. In addition, a meta-analysis of clinical trials of antibiotic therapy in patients with coronary artery disease (CAD) failed to demonstrate any benefit with regard to the mortality or cardiovascular events in CAD patients. This result suggests that gut microbiota modification by antibiotics does not modify the evolution of CAD [30]. In another study, 4,012 patients with documented stable CAD received either 600 mg of azithromycin or placebo weekly for 1 year, with no effect on the risk of cardiac events among patients with stable CAD [31].

Nevertheless, in a Dahl S rat model of ischemia/reperfusion injury of the heart, microbiota was shown to increase the severity of myocardial infarction. In fact, the authors showed that treatment with vancomycin, a very poorly absorbable antibiotic, led to a 27 % reduction in myocardial infarction and a 35 % increase in postischemic mechanical function recovery. This effect was associated with a change in the gut microbiota at both the bacterial and fungal levels and to reduced plasma levels of leptin. The latter datum was confirmed mechanistically by administration of the leptin-suppressing probiotic *Lactobacillus plantarum* 299v, which resulted in a 29 % reduction in myocardial infarction [32]. These first two contradictory examples of antibiotic utilization (azithromycin vs vancomycin) clarify the complexity of gut microbiota-based intervention in terms of efficacy and properties of the applied protocol. Interestingly, *L. plantarum* PH04 (another strain of the aforementioned probiotic) also was described as a cholesterol-lowering probiotic in hypercholesterolemic mice, in which administration of this probiotic was associated with a 10-fold increase in fecal lactic acid bacteria [33].

**Fig. 1:**
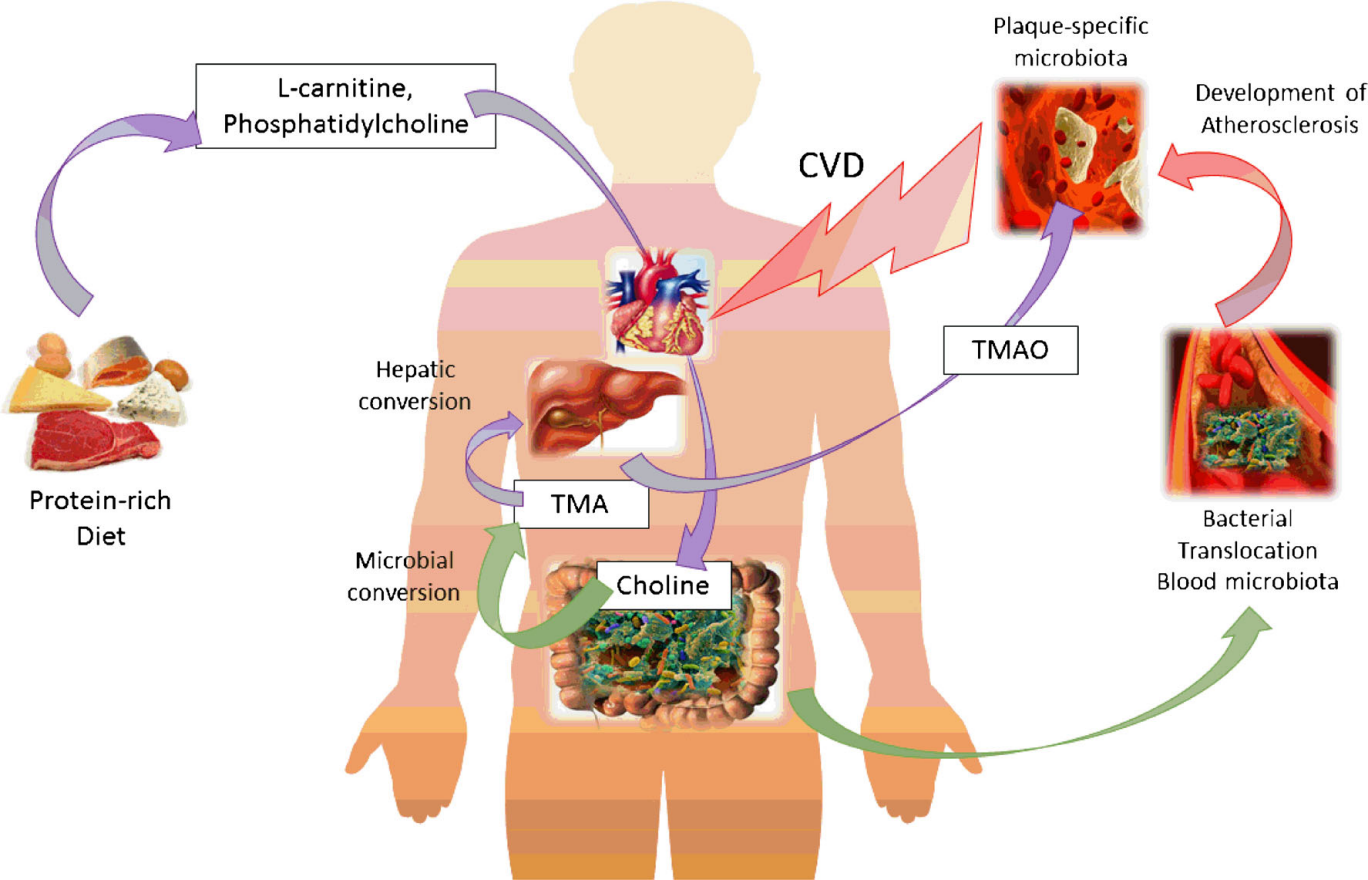
Gut microbiota and its impact on the cardiovascular system. Products from a protein-rich diet, such as L-carnitine and phosphatidylcholine, may be metabolized into choline, which is converted to trimethylamine (*TMA*) by the gut microbiota. TMA may be oxidized into the liver to form TMA-*N*-oxide (*TMAO*), which can promote the formation of atherosclerotic plaque [28••]. On the other hand, diet-induced gut microbiota dysbiosis may result in bacterial translocation into the systemic blood flow, where a blood microbiota (almost 90 % Gram-negative bacteria) may become established [40••]. Subsequently, atherosclerotic plaques may develop and promote atherosclerosis and cardiovascular diseases. Interestingly, a plaque-specific blood-like microbiota exists, and it is dominated by Gram-negative bacteria (*Proteobacteria* phylum) as well [39]

Recently, several works highlighted a new mechanism by which the intestinal microbiota seems to participate in the etiology of CVD. This discovery is based on metabolomic studies of plasma samples from patients with atherosclerotic plaque formation. The authors found that the concentration of the metabolite TMAO was elevated in patients with atherosclerosis and was correlated directly with this pathology. Moreover, the authors demonstrated that the gut microbiota is responsible for TMAO synthesis by converting choline, an essential nutrient, into TMA. For example, bacteria from the class Erysipelotrichia (phylum Firmicutes) reportedly can metabolize choline to TMA [34], hence mimicking a choline deficiency. Next, TMA is absorbed and rapidly oxidized by hepatic cells to form TMAO [35], which is responsible for macrophage foam cell formation, as reported in Fig. 1, by reducing reverse cholesterol transport in vivo, and consequently promotes cholesterol accumulation in the foam cells of atheroma [28••]. However, the molecular mechanisms by which TMAO reduces reverse cholesterol transport are not well understood.

Therefore, on one hand, bacteria from the class Erysipelotrichia may promote atherosclerosis via TMA–TMAO production [36]. On the other hand, these bacteria may promote nonalcoholic fatty liver disease (NAFLD) by reducing choline availability for the synthesis of very low-density lipoprotein in the liver, resulting in triglyceride accumulation in the hepatocytes [37]. It is noteworthy that the abundance of bacteria from the class Erysipelotrichia also increases with an iron-rich diet, which promotes gut epithelial cell stress via iron accumulation in the enterocytes and, consequently, inflammation-induced dysbiosis of the gut microbiota. Hence, an iron-rich diet may be directly responsible for the development of NAFLD and atherosclerosis via alteration of the gut microbiota [36].

Besides the intestine, several other organs of the body, such as the mouth, skin, vagina, and airways, harbor their own microbiota, each with a specific bacterial proportion [38]. Interestingly, atherosclerotic plaque also has been shown to harbor its own microbiota, dominated by members of the phylum Proteobacteria (e.g., *Escherichia coli*) [39]. Remarkably, this phylum is also the most abundant of the newly identified blood microbiota we discovered recently in diabetic patients in the Epidemiological Study on the Insulin Resistance Syndrome (DESIR) cohort [40••]. Therefore, it may be speculated that the establishment of blood microbiota might represent the first step in the kinetics of atherosclerotic plaque formation.

Bacteria from the genus *Collinsella* also were found to be enriched in patients with symptomatic atherosclerosis, defined as the presence of stenotic atherosclerotic plaques at the level of the carotid artery and leading to cerebrovascular events. By contrast, bacteria belonging to *Roseburia* and *Eubacterium* were augmented in the gut microbiota of healthy controls [41]. In this study, by Karlsson et al. [41], the microbiome (functional gene level) also was investigated, revealing enrichment of genes encoding for peptidoglycan, among others, in the metagenome from patients.

Among the strategies to target the gut microbiota, prebiotics [42, 43] and probiotics [44] are those used most often. In an article published in 2014, Gan et al. proposed using *Lactobacillus rhamnosus* GR-1 as a probiotic for treating heart failure in rats subjected to 6 weeks of sustained coronary artery occlusion. The authors showed that the probiotic treatment could delay heart failure development after coronary occlusion in rats. However, the probiotic treatment did not affect the cecal microbiome profile [45].

In another article, the authors showed that *Methanomassiliicoccus luminyensis* B10, a methanogen bacterium, can deplete TMA by reducing it with H_2_ for methanogenesis. Therefore, they suggest that bacteria from this group may be used as probiotics to treat metabolic disease as well as CVD by reducing TMA and, hence, TMAO plasma levels [46]. In this context, plasma levels of choline, as well as betaine, may be used as a prognostic value to determine the risk of developing a major adverse cardiac event (MACE), such as death, myocardial infarction, or stroke [47]. In this study, Wang et al. [47] examined a 3-year survey of 3,903 sequential stable subjects who underwent elective diagnostic coronary angiography. However, the authors reported that values of choline and betaine are prognostic of MACE only in association with increased TMAO plasma levels.

Besides CVD in adulthood, this disease also represents a serious health challenge in term infants. Congenital heart disease (CHD) is among the first causes of premature death in the first year of life. CHD also is one of the most important risk factors for necrotizing enterocolitis (NEC) in term infants. Probiotic treatment with *Bifidobacterium longum* subsp. *infantis* is reported to be effective in terms of reducing inflammation in NEC, even if the gut microbiota appears not to be significantly affected by the probiotic [48]. Interestingly, CHD also is associated with dysbiosis of the gut microbiota, such as reduced total bacteria, Bacteroidetes, and bifidobacteria, when healthy infants are compared with CHD infants. Nevertheless, administration of the probiotic *Bifidobacterium infantis* again did not show a significant change in plasma cytokines or in the microbial stool profiles of CHD infants compared with the placebo group [49].

## Periodontal Diseases, Oral Microbiota Translocation, and CVDs

Periodontitis (also known as periodontal disease [PD]) is an inflammatory disease of the oral cavity defined as a chronic bacterial infection of the soft and hard tissues supporting and surrounding the teeth and caused by Gram-negative capnophilic bacteria [50, 51].

Besides metabolic diseases such as type 2 diabetes and obesity, PD also is considered a risk factor for CVD. In fact, PD has been associated with CVD and atherosclerosis [52], suggesting that the oral microbiota may be causal in the etiology of atherosclerosis. In this context, we recently reported that 1-month colonization with periodontal pathogens such as *Porphyromonas gingivalis*, *Prevotella intermedia*, and *Fusobacterium nucleatum* aggravated high-fat diet (HFD)-induced metabolic alteration (glucose intolerance) as well as systolic and diastolic arterial pressure in diabetic mice [53]. The molecular mechanism underlying this phenotype is associated with Gram− bacterial lipopolysaccharide (LPS), because we also showed that CD14 knock-out mice were protected from HFD-induced periodontal defects [54].

The possibility that LPS might bridge PD and CVD also was suggested by another paper showing that LPS from *Porphyromonas gingivalis* was responsible for inflammation-induced CVD via increased oxidative stress and mitochondrial dysfunction [55].

Oral bacterial spread into the bloodstream (bacterial translocation) also may be the origin of PD-induced endocarditis and myocardial and/or cerebral infarction, especially in patients with heart valve dysfunction, as the result of uncontrolled bacteremia [56]. For example, oral streptococci have been found to dominate infective endocarditis [57]. Following bacterial translocation, Calandrini et al. [58] detected the periopathogen *Aggregatibacter actinomycetemcomitans* in 20 % of endarterectomy (the surgical removal of atherosclerotic plaque from an artery) specimens from patients with myocardial infarction, followed by *Pseudomonas* species. However, metagenomic analysis of the oral microbiota from 15 patients with clinical atherosclerosis failed to show differences when compared with control subjects [39]. In addition, the same study failed to demonstrate that atherosclerosis patients harbor a gut microbiota different from that of control subjects.

## Conclusions

Currently, the worldwide scientific community is paying more attention to the putative consequences of alterations in the gut microbiota and the progression of cardiometabolic disease, given the pleiotropic range of host functions that may be affected by gut microbiota dysbiosis [59, 60, 16]. Despite convincing studies showing the deleterious effects of gut microbiota metabolism of a protein-rich diet, gut microbiota metabolism of L-carnitine, and the association with cardiovascular risk, controversy persists. In fact, it also has been shown that L-carnitine may ameliorate metabolic diseases by increasing insulin sensitivity of the skeletal muscle and may reduce ischemic heart disease. Moreover, dietary fish is rich in TMAO, despite the beneficial effects of fish oil on cardiovascular events [61].

These results demonstrate that the complex ecology of the gut microbiota and its metabolic behavior must be considered as being associated with specific metabolic conditions, rather than generalized as a common mechanism of etiology. Therefore, uncertainty remains as to whether the gut microbiota should be considered a cause or consequence of a given pathology.

The path to finding and developing microbe-based therapies passes ineluctably through the elucidation of this relevant dilemma.
